# Inhibition of Small Conductance Calcium-Activated Potassium (SK) Channels Prevents Arrhythmias in Rat Atria During β-Adrenergic and Muscarinic Receptor Activation

**DOI:** 10.3389/fphys.2018.00510

**Published:** 2018-06-05

**Authors:** Lasse Skibsbye, Anne K. Bengaard, A. M. Uldum-Nielsen, Kim Boddum, Torsten Christ, Thomas Jespersen

**Affiliations:** ^1^Cardiac Physiology Laboratory, Department of Biomedical Sciences, Faculty of Health and Medical Sciences, University of Copenhagen, Copenhagen, Denmark; ^2^Department of Experimental Pharmacology and Toxicology, University Medical Center Hamburg-Eppendorf, DZHK: German Centre for Cardiovascular Research, Hamburg, Germany

**Keywords:** atrial fibrillation, SK channels, parasympathetic and sympathetic activation, β-adrenergic and muscarinic activation, action potential recordings

## Abstract

Sympathetic and vagal activation is linked to atrial arrhythmogenesis. Here we investigated the small conductance Ca^2+^-activated K^+^ (SK)-channel pore-blocker *N*-(pyridin-2-yl)-4-(pyridine-2-yl)thiazol-2-amine (ICA) on action potential (AP) and atrial fibrillation (AF) parameters in isolated rat atria during β-adrenergic [isoprenaline (ISO)] and muscarinic M2 [carbachol (CCh)] activation. Furthermore, antiarrhythmic efficacy of ICA was benchmarked toward the class-IC antiarrhythmic drug flecainide (Fleca). ISO increased the spontaneous beating frequency but did not affect other AP parameters. As expected, CCh hyperpolarized resting membrane potential (-6.2 ± 0.9 mV), shortened APD_90_ (24.2 ± 1.6 vs. 17.7 ± 1.1 ms), and effective refractory period (ERP; 20.0 ± 1.3 vs. 15.8 ± 1.3 ms). The duration of burst pacing triggered AF was unchanged in the presence of CCh compared to control atria (12.8 ± 5.3 vs. 11.2 ± 3.6 s), while β-adrenergic activation resulted in shorter AF durations (3.3 ± 1.7 s) and lower AF-frequency compared to CCh. Treatment with ICA (10 μM) in ISO -stimulated atria prolonged APD_90_ and ERP, while the AF burden was reduced (7.1 ± 5.5 vs. 0.1 ± 0.1 s). In CCh-stimulated atria, ICA treatment also resulted in APD_90_ and ERP prolongation and shorter AF durations. Fleca treatment in CCh-stimulated atria prolonged APD_90_ and ERP and abbreviated the AF duration to a similar extent as with ICA. Muscarinic activated atria constitutes a more arrhythmogenic substrate than β-adrenoceptor activated atria. Pharmacological inhibition of SK channels by ICA is effective under both conditions and equally efficacious to Fleca.

## Introduction

Atrial fibrillation is the most prevalent cardiac arrhythmia, associated with increased cardiac morbidity and mortality (Andrade et al., 2014; Jalife and Kaur, 2015; Walfridsson et al., 2015). Due to inadequate efficacy and adverse events of current treatments, there exists a high unmet medical need for efficacious and safe antiarrhythmic drug (AAD) therapy. However, in order to find and exploit potential new AF targets, a more thorough mechanistic understanding of arrhythmogenesis and progression of AF is needed (Roy et al., 2000; Ravens et al., 2013; Wettwer et al., 2013).

Well-balanced autonomic nervous regulation is important for adjusting and upholding proper cardiac electrophysiology. However, at the same time, increased activity of both vagal and sympathetic activation and autonomic imbalance is believed to trigger and maintain supraventricular arrhythmias such as AF (Chen et al., 2014; Heijman et al., 2016; Hou et al., 2016; Östenson et al., 2017). Furthermore, autonomic receptors have been described as an efficacious clinical target for interventional anti-AF therapy (Linz et al., 2014). Autonomic nerves exert stimulatory or inhibitory effects via adrenergic endogenous neurotransmitter norepinephrine (NE) and muscarinic acetylcholine (ACh) receptors, respectively (Christ et al., 2014). Sympathetic nerves exert their effect through various α- and especially β-adrenergic receptor (β-AR) subtypes. β1 receptors, activating through cAMP and phosphorylation by protein kinase A (PKA), have a profound effect on both calcium handling and electrophysiology of atrial and ventricular cardiomyocytes (Brodde et al., 2001; Volders, 2010). The cholinergic muscarinic receptor includes M_1-5_ subtypes, with type-2 muscarinic subtype (M_2_) highly expressed in the atria, SA- and AV-node (Brodde et al., 2001; Linz et al., 2014). The cardiac M_2_ receptor is an inhibitory Gα_i_ subtype, activating I_K,ACh_, composed of Kir3.1 (*GIRK1*) and Kir3.4 (*GIRK4*) subunits (Krapivinsky et al., 1995). Activation of I_K,ACh_ results in RMP hyperpolarization, abbreviated atrial APD, and effective refractory period (Shimizu et al., 1994; Wang et al., 2013).

Several studies have reported that the voltage-insensitive SK channel constitutes a potential atrial selective target for AF treatment (Xu et al., 2003; Tuteja et al., 2005; Ozgen et al., 2007; Li et al., 2009; Diness et al., 2010, 2011). In the heart, SK channels are found predominantly expressed in the atria in several species, including rats and humans (Diness et al., 2010; Skibsbye et al., 2014). Pharmacological blockage of SK channels in acutely induced AF animal models, ranging from rat to horse, has shown profound antiarrhythmic properties (Diness et al., 2010, 2015; Haugaard et al., 2015; Skibsbye et al., 2015). Furthermore, studies in human atrial trabeculae muscle from patients in sinus rhythm (SR) reported SK channels to participate in atrial repolarization and that pharmacological SK channel blockage prolongs APD and refractoriness, and depolarize the atrial RMP (Skibsbye et al., 2014).

When investigating the electrophysiological properties during AF, it is challenging to obtain accurate intracellular voltage potential measurements due to shivering and ridged movement of the atrial muscle. However, recently, we developed an *ex vivo* rat atrial model, using explanted beating atria, in which transmembrane AP measurements can be recorded continuously at initiation and during episodes of AF without loss of intracellular recording (Skibsbye et al., 2015). Compared to baseline AP stimulation, recordings during paroxysmal and persistent tachy-arrhythmias revealed the minimal diastolic (take-off) potential to be depolarized ≈10–15 mV and AP amplitude, as well as upstroke velocity, to be drastically reduced as a consequence of increased sodium channel inactivation at depolarized potentials. This technique thereby constitutes a highly interesting model for addressing AP parameter changes following muscarinic and β-AR stimulation in the context of atrial arrhythmias and a utility for mechanistically understanding the electrophysiological effects following pharmacological interventions.

In the present study, we pursue two objectives: (i) investigate the acute effect on electrophysiological parameters following sympathetic and parasympathetic pharmacological activation in the context of arrhythmia and (ii) benchmarking the antiarrhythmic efficacy of the small molecule tool compound ICA, which is a pore-blocker of SK channels, with a relatively high selectivity over other cardiac ion channels (Skibsbye et al., 2014) toward Fleca, a classical class IC sodium channel inhibitor used for rhythm control in AF treatment (Apostolakis et al., 2013) in this mechanistic AF model.

## Materials and Methods

Experiments were performed under a license from the Danish Ministry of Justice (License No. 2012-15-2934) and in accordance with the Danish Guidelines for Animal Experiments according to the European Commission Directive 86/609/EEC.

### Microelectrode Measurement of Rat Right Atrium

Adult male Sprague Dawley (Crl:SD) rats (Taconic, Cologne, Germany) weighing between 345 and 575 g were used. All experiments were conducted using a refined AF model of isolated rat right atrium described recently (Skibsbye et al., 2015). The isolated rat right atrium was placed in a Steiert papillary muscle bath (Hugo Sachs Harvard Apparatus GmbH, March, Germany) and perfused with Tyrode’s solution at 37°C contain (in mM): NaCl 118.1, KCl 4.0, CaCl_2_ 1.8, MgCl_2_ 1.0, NaH_2_PO_3_ 24.9, and D-glucose 11.0, bubbled with 95% O_2_ and 5% CO_2_. Atrial APs were recorded with regular sharp capillary borosilicate glass microelectrodes (Warner capillary glass, Harvard Apparatus UK), pulled on a Sutter Instrument Co. Model P-97). Recording electrodes acquired a tip resistance of 10–30 MΩ when filled with 3.0 M KCl. The AP recordings were obtained with acquisition software LabChart 7 Pro (AD Instruments, Sydney, Australia).

### β-Adrenergic and Muscarinic Receptor Activation

**FIGURE 1:**
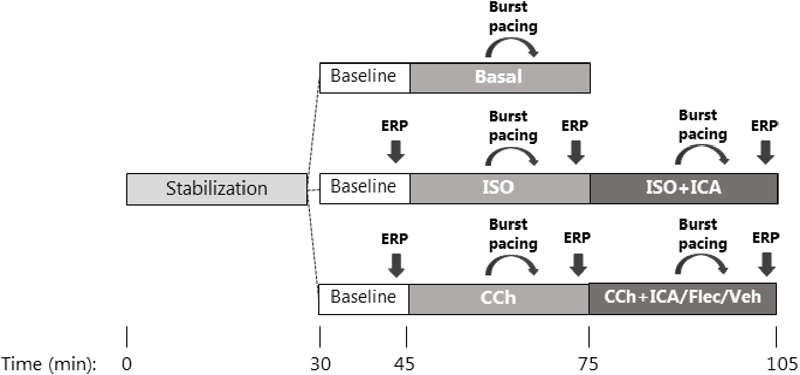
Flowchart. Graphic overview of the experimental design. After mounting of the right atrium in an organ bath and initiation of stimulated pacing, all experiments were allowed to stabilize for 30 min. Experiments were divided into three randomized sub-studies. **(Top panel)** Basal characterization of AP parameters without any premedication or antiarrhythmic treatment followed by induction of consecutive runs of AF by programmed atrial burst pacing. **(Middle panel)** Baseline AP recordings followed by pre-treatment with [ISO], AP characterization, and AF induction followed by antiarrhythmic treatment with [ICA]. **(Lower panel)** Baseline AP recordings followed by pre-treatment with [CCh], AP characterization, and AF induction before antiarrhythmic treatment with either [ICA], [Fleca], or [vehicle] with the purpose to benchmark the antiarrhythmic efficacy between the two drugs in the muscarinic activated atria.

A graphic overview of the experimental setup is given in **Figure 1**: atrial pacing was performed at double threshold current (rheobase; usually 0.01–0.03 mA at the beginning of the experiment and before every aERP measurement) with a stimulus duration (pulsewidth) of 2 ms. Following electrode impalement and satisfactory baseline AP recordings, the right atrium was allowed to stabilize for 30 min. Inclusion criteria for satisfactory AP recordings were RMP ≈-75 mV and amplitude ≈ 85 mV. Of note, the success rate for obtaining stable impalements in the ISO-treated atria was low due to rigorously beating, disrupting the seal. Following a 15 min baseline recording, the atrium was exposed to either 1 μM ISO or 1 μM CCh or Ringer without drugs (Basal). Under control condition, the isolated rat right atrium had a spontaneous beating frequency of 3.5–4.5 Hz. Thus, in order to pace above the intrinsic rate, all experiments (besides those with ISO application) were performed at a 5 Hz pacing frequency. ISO increased the spontaneous atrial rate, wherefore these experiments were performed at 8 Hz pacing frequency. After 30 min of superfusion with ISO 1 μM or CCh 1 μM either of the test compounds [ICA 10 μM, Fleca 1 μM or vehicle dimethyl sulfoxide (DMSO)] were applied to the buffer.

### Atrial Effective Refractory Period Measurement

Atrial effective refractory period measurements were performed using an S1–S2 stimulation protocol, by decreasing the S1–S2 interval by 2 ms increments with aERP defined as the longest S1–S2 interval, which was unable to initiate an extra S2-mediated AP.

### Arrhythmia Induction

Atrial fibrillation was induced by a burst pacing protocol of 50 Hz using double rheobase voltage threshold for approximately 1 s repeated up to five times, depending on whether the burst initiated an arrhythmia or not. When an arrhythmia was initiated, stimulation was stopped until the arrhythmia spontaneously reverted into SR or until the duration of arrhythmia lasted more than 120 s. If the arrhythmia lasted more than 120 s, electrical cardioversion was performed by single bursts of high-voltage stimulation. The burst pacing induction protocol was applied in the presence of ISO or CCh or basal stimulation 20–25 min after application and after 30 min incubation with either ICA, Fleca, or DMSO, serving as time-matched control (TMC). AF duration was calculated as mean of all consecutive arrhythmic periods following burst pacing, including zero counts when there was no AF induction.

### Chemicals

The compounds applied were of analytical grade with CCh (carbamoylcholine chloride), ISO (ISO hydrochloride), and Fleca (Fleca acetate) acquired from Sigma-Aldrich (Steinheim, Germany). ICA was synthesized at NeuroSearch A/S (Ballerup, Denmark). CCh and ISO were dissolved in Milli Q water, whereas ICA and Fleca were dissolved in DMSO (anhydrous ≥ 99.9%).

### Data and Statistical Analysis

**FIGURE 2:**
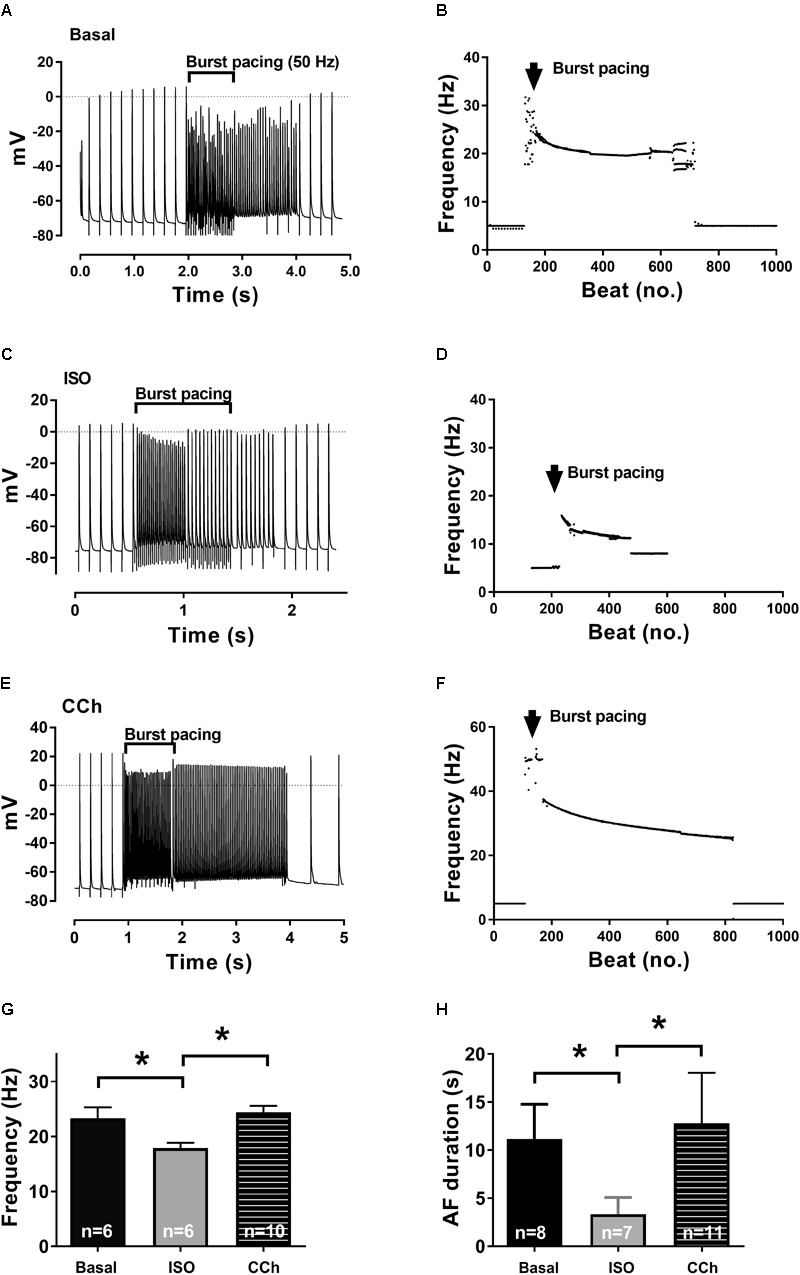
Isoprenaline and carbachol (CCh) effects on frequency and AF duration. Recordings in the presence of no activators (basal, **A**), [ISO] (β-adrenergic, **C**), and [CCh] (muscarinic, **E**) activation. Burst pacing of the isolated right atrium leads to AF episodes of various lengths, all reverting spontaneously back to SR. Short (1–2 s) runs of AF are displayed to exemplify both initiation and termination of AF. **(B,D,F)** Diary plots of beating rate (1000 beats) from representative experiments, depicting the frequency during the induction and the free run of an arrhythmia before reverting back to SR. **(G)** AP frequencies in the entire group (mean ± SEM). **(H)** AF duration in the absence of [ISO] or [CCh] (basal) or in the presence either [CCh] or [ISO]. Statistical analysis was done using one-way ANOVA, comparing basal, CCh, and ISO. ^∗^*p* < 0.05.

**FIGURE 3:**
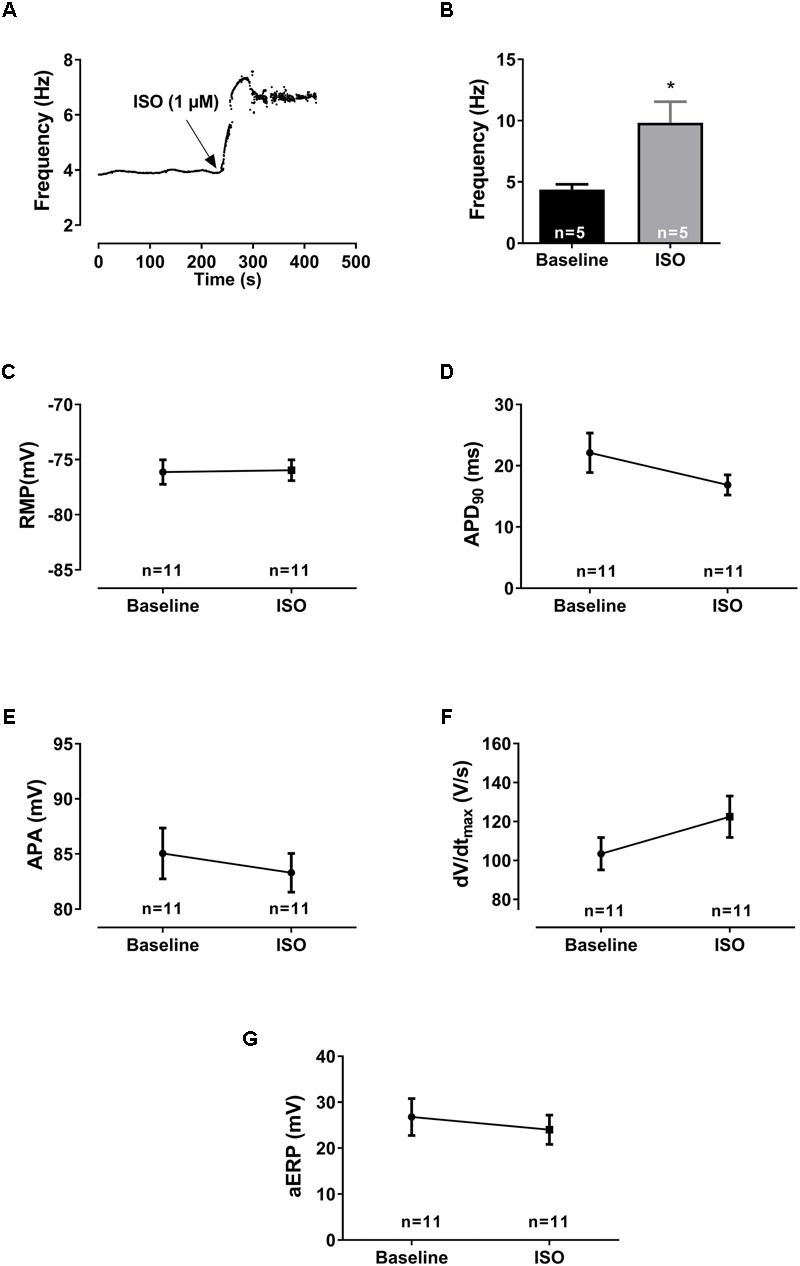
Electrophysiological effects of [ISO]. **(A)** Diary plot depicting the effect of β-adrenergic activation on the pacemaker frequency (after application of [ISO] in an isolated rat right atrium). An immediate increase in intrinsic beating frequency was observed shortly after application of [ISO] to the perfusion buffer. After a transient peak, the frequency was maintained over time. **(B)** Bar graph shows the mean values ± SEM (*n* = 5) during basal intrinsic frequency and after [ISO] application. The effect of β-adrenergic activation on AP parameters (*n* = 9): **(C)** the resting membrane potential (RMP), **(D)** action potential duration (APD_90_), **(E)** amplitude (APA), **(F)** upstroke velocity (*dV*/*dt*_max_), and **(G)** atrial effective refractory period (aERP), all measured before and after application of 1 μM [ISO] at a stimulated rate of 8 Hz. The calculated AP parameters were averaged from 10 consecutive APs before aERP measurements for each experiment. Statistical analysis was done using a Student’s paired *t*-test. ^∗^*p* < 0.05.

**FIGURE 4:**
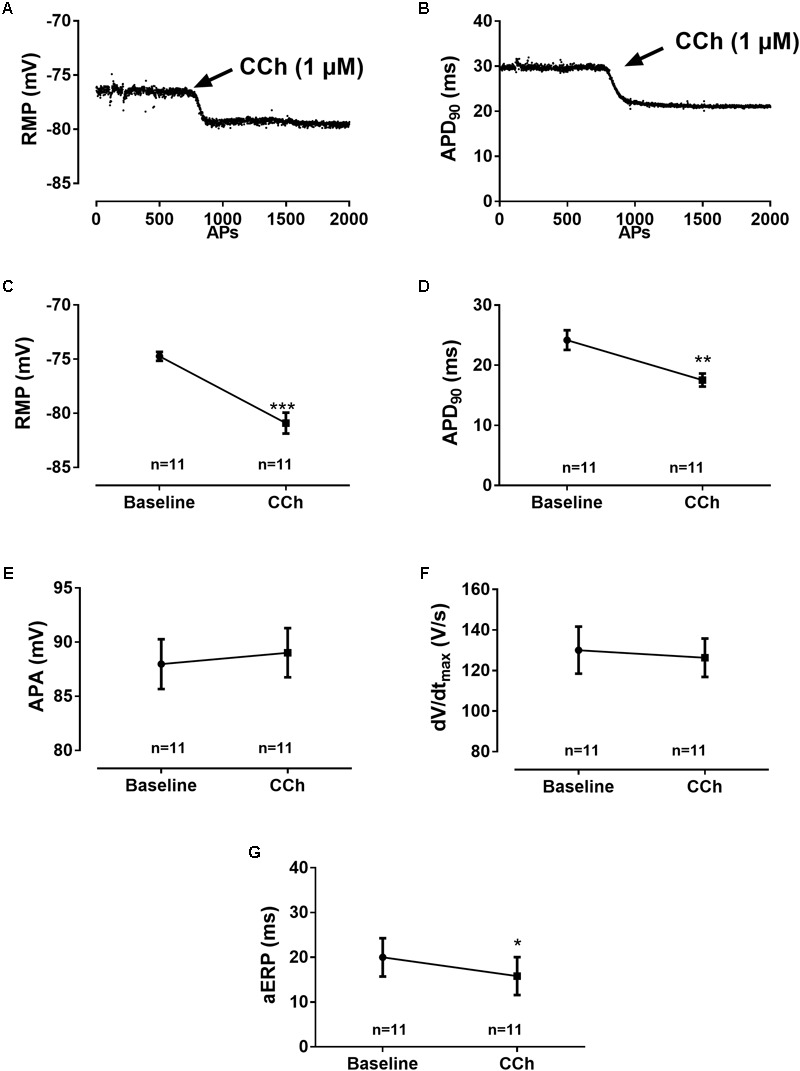
Electrophysiological effects of [CCh]. Diary plots from an experiment, 0–5 min after 1 μM [CCh] was applied, depicting the time dependence of [CCh] effects on **(A)** RMP and **(B)** APD_90_ as a function of action potentials (APs). The initial to full effect of CCh is abrupt and happens within 100–200 APs. Effect of muscarinic activation on AP parameters (*n* = 11): **(C)** Average of the resting membrane potential (RMP), **(D)** action potential duration at 90% repolarization (APD_90_), **(E)** action amplitude (APA), **(F)** maximum upstroke velocity (*dV*/*dt*_max_), and **(G)** atrial effective refractory period (aERP), all measured before and after adding 1 μM CCh. Statistical analysis was done using a Student’s paired *t*-test. ^∗^*p* < 0.05, ^∗∗^*p* < 0.005, and ^∗∗∗^*p* < 0.001.

**FIGURE 5:**
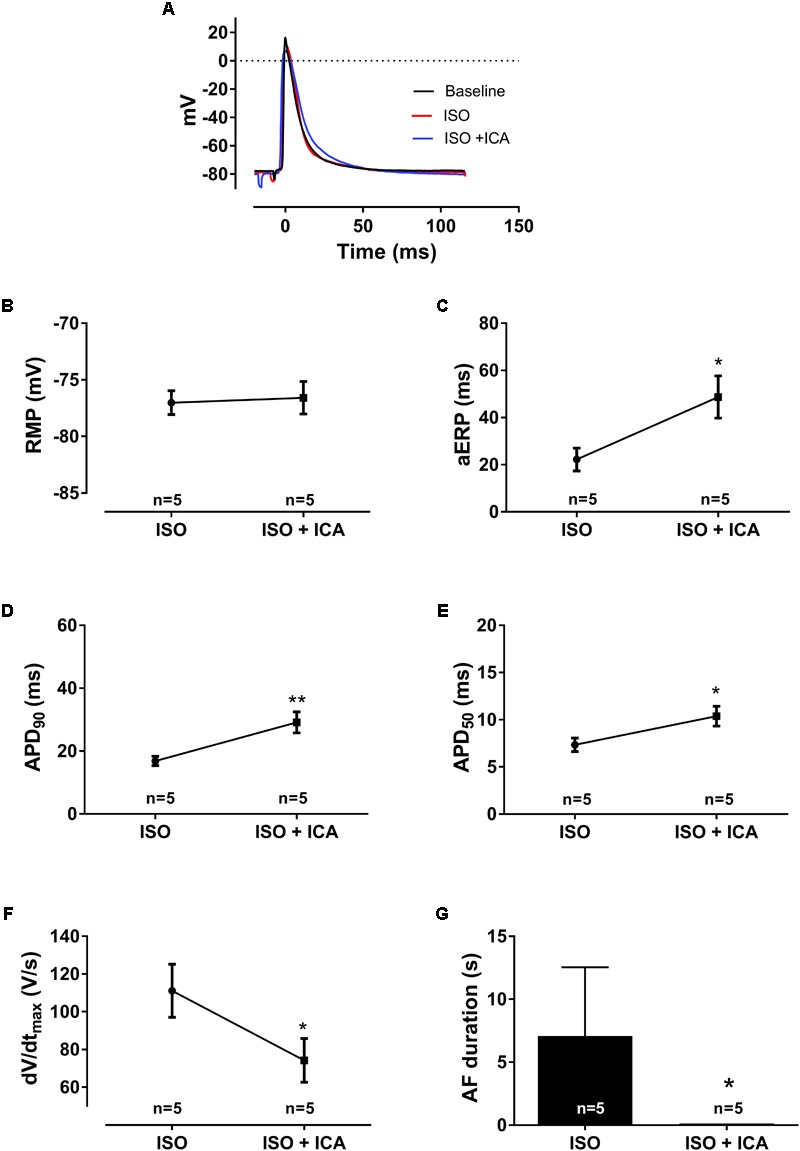
Electrophysiological effects of SK channel inhibition with [ICA] on [ISO] pre-stimulated atria (1 μM [ISO], 10 μM [ICA], *n* = 5). **(A)** Representative traces of action potentials. **(B)** Resting membrane potential (RMP), **(C)** atrial effective refractory period (aERP), **(D)** action potential duration at 90% repolarization (APD_90_), **(E)** action potential duration at 50% repolarization (APD_50_), **(F)** upstroke velocity (*dV*/*dt*_max_), and **(G)** AF duration (s), at pacing rate 8 Hz. The significance level was found using a two-way ANOVA by comparing the average of 10 consecutive APs before aERP measurements at baseline and after application of drug. ^∗^*p* < 0.005 and ^∗∗^*p* < 0.01.

**FIGURE 6:**
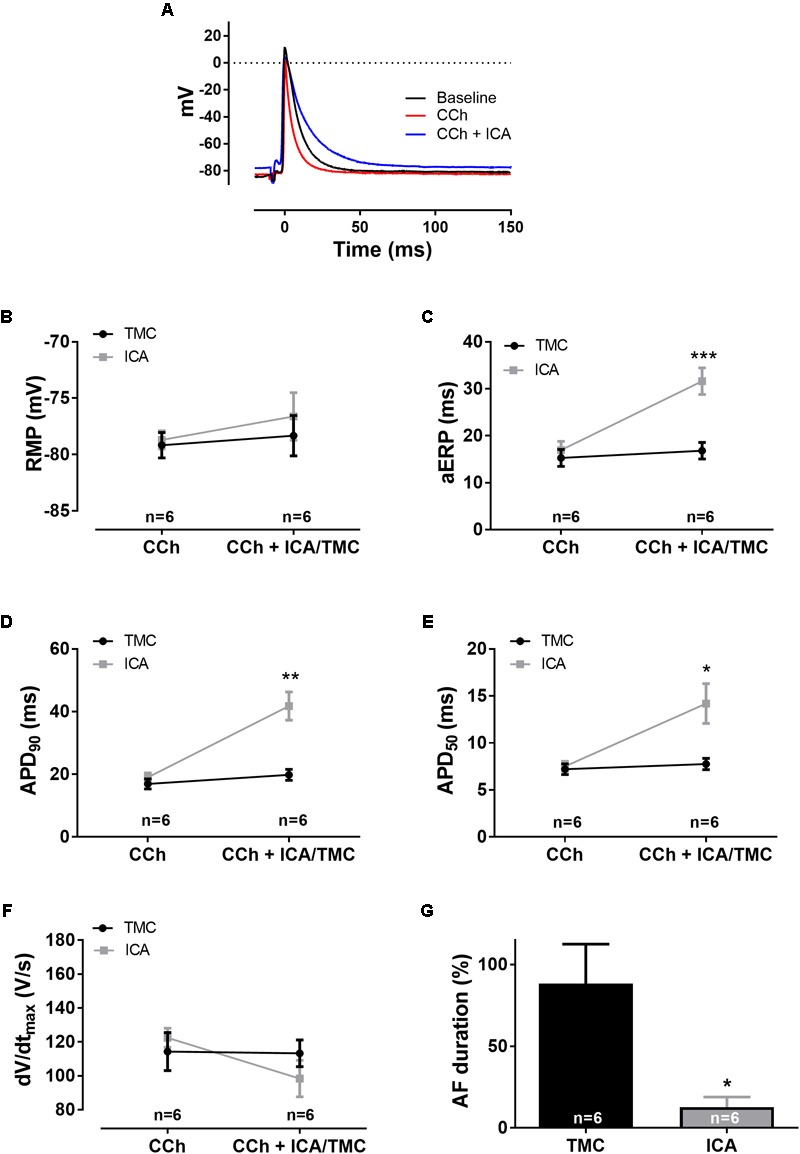
Electrophysiological effects of SK channel inhibition with [ICA] on [CCh] pre-stimulated atria (1 μM [CCh], 10 μM [ICA], *n* = 12). **(A)** Representative traces of action potentials. **(B)** Resting membrane potential (RMP), **(C)** atrial effective refractory period (aERP), **(D)** action potential duration at 90% repolarization (APD_90_), **(E)** action potential duration at 50% repolarization (APD_50_), **(F)** upstroke velocity (*dV*/*dt*_max_), and **(G)** data on AF duration normalized to baseline (%), at pacing rates of 5 Hz. The significance level was found using a two-way ANOVA by comparing the average of 10 consecutive APs before each aERP measurement at baseline and after application of drug or vehicle. ^∗^*p* < 0.05, ^∗∗^*p* < 0.005, and ^∗∗∗^*p* < 0.001.

**FIGURE 7:**
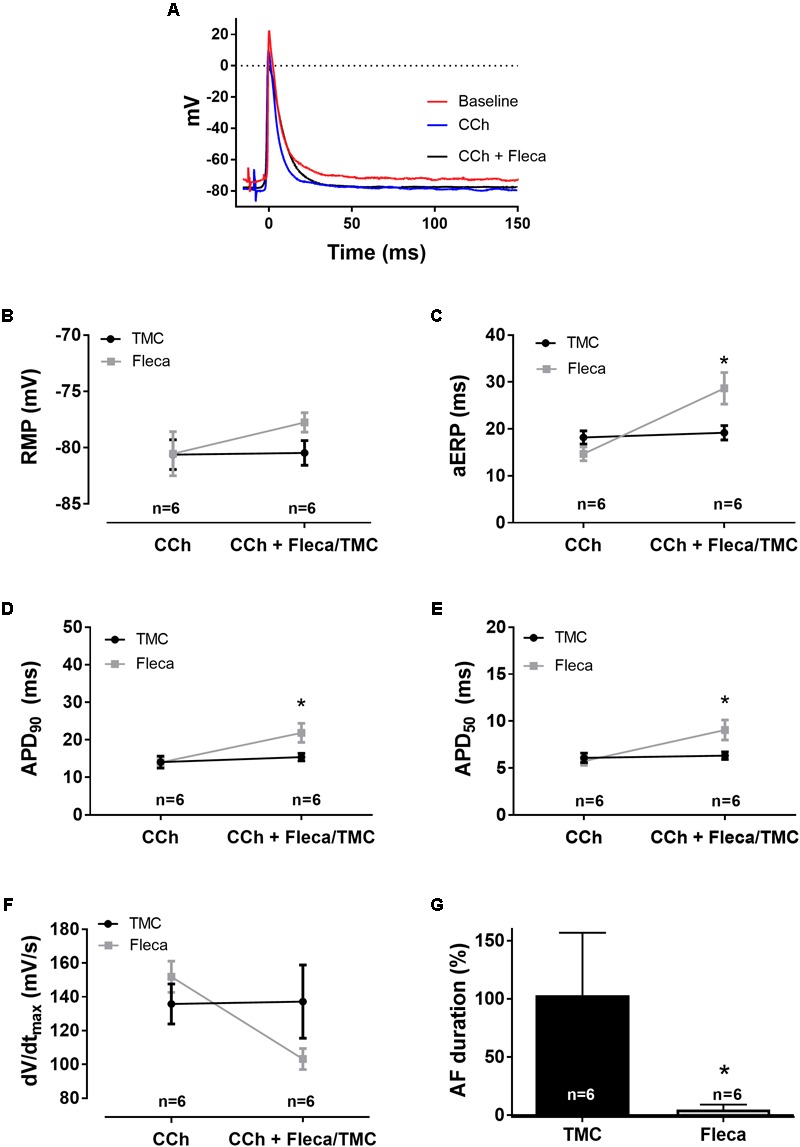
The effect of flecainide on CCh-pre-stimulated atria (1 μM [CCh], 1 μM [flecainide], *n* = 12). **(A)** Representative traces of action potentials. **(B)** Resting membrane potential (RMP), **(C)** atrial effective refractory period (aERP), **(D)** action potential duration at 90% repolarization (APD90), **(E)** action potential duration at 50% repolarization (APD50), **(F)** upstroke velocity (*dV*/*dt*_max_), with the time-matched control group being unchanged (from 136 ± 12 to 137 ± 22 mV/s) and the flecainide group being reduced (from 152 ± 9 to 103 ± 6 mV/s), and **(G)** data on AF duration normalized to baseline (%), at pacing rates of 5 Hz. The significance is calculated using a two-way ANOVA by comparing the average of 10 consecutive APs before each aERP measurement at baseline and after application of drug or vehicle. ^∗^*p* < 0.05, ^∗∗^*p* < 0.005, and ^∗∗∗^*p* < 0.001.

Data acquisition was performed using LabChart 7-Pro software (ADInstrument, Dunedin, New Zealand). For data analysis and drawings, GraphPad Prism 6 software (GraphPad Software, San Diego, CA, United States) was used. All data are presented as mean ± standard error of the mean (SEM). For calculations of AP parameters, 10 consecutive APs were averaged. In all statistical analyses, the level of significance was 95% confidence interval, which means *p*-values < 0.05 were considered statistically significant. For statistical analysis, two-tailed Student’s paired *t*-test was applied to data in **Figures 3, 4**. One-way analysis of variance (ANOVA) was applied to data in **Figure 2**. Two-way ANOVA with Bonferroni *post hoc* test for multiple comparisons was applied to data in **Figures 5–7**. Statistical analysis of antiarrhythmic efficacy between Fleca and ICA was done using Student’s unpaired *t*-test.

## Results

Interrogation of β-adrenergic and muscarinic activation on atrial APs in isolated right rat atrium was performed based on a newly developed AF model in isolated rat atria (Skibsbye et al., 2015). In this model, a high-resistance electrode is impaled into a single cardiomyocyte in a multicellular tissue, making it possible to measure transmembrane APs in beating isolated rat atria. The β-AR agonist ISO (1 μM) and the muscarinic receptor agonist CCh (1 μM) were used to mimic sympathetic and vagal activation, respectively. The atria were stimulated at 5 Hz pacing in all experiments apart from the ISO-treated group which were stimulated at 8 Hz to overrule the increase in intrinsic beating frequency. After 30 min of stabilization and a 15 min baseline period, tissues were randomized to application of ISO, CCh, or TMC (basal; **Figure 1**). Following 30 min of application of ISO, the SK channel inhibitor ICA was added for another 30 min. In the experiments mimicking vagal activation by the use of CCh, the tissue was following the 30 min incubation challenged by ICA, Fleca, or DMSO (vehicle). aERP was measured by an S1–S2 stimulation protocol at the end of each period in the ISO and CCh groups. Short burst pacing periods (50 Hz, ∼1000 ms at double voltage threshold) were applied to evoke runs of arrhythmia which had a mean duration of ≈10 s. The “basal” group was conducted to compare the AF duration between non-treated atria and ISO and CCh treated atria. The remaining of experiments in this study were devoted to addressing AP parameters, ERP, and AF and were performed as paired experiments with either ISO or CCh activated atria including TMC groups.

Examples of runs of AF following control (basal), muscarinic (CCh), and β-adrenergic (ISO) activation are shown in **Figure 2**. Arrhythmias transiently had a high dominant frequency between 20 and 40 Hz; however, after a few seconds and especially during longer AF runs, the frequency declined reaching an almost steady-state frequency before spontaneous reversion to SR (**Figures 2B,D,F**). These observations correspond well with previous recordings performed in non-stimulated atria (Skibsbye et al., 2015).

Interestingly, ISO-stimulated atria had significant shorter AF durations than both control (basal) and CCh activated atria, and the AF frequency prior to the reversion to SR was significantly lower in the ISO group. While the CCh activated atria exhibited AF runs more consistently than in the control group (with high transient frequencies), the difference in AF duration and frequency did not reach statistical significance. During the AF episodes, the minimum diastolic potential [measured just prior to the next AP (take-off potential)] was depolarized to ≈-65 mV consistently in all three groups.

### Effects of β-Adrenergic Stimulation on Action Potential Parameters

Isoprenaline application induced a fast increase in intrinsic SR frequency (from 4.4 to 9.8 Hz, transiently) and stabilizing at ≈7 Hz after a few minutes before stimulated pacing at 8 Hz was applied (**Figures 3A,B**). At a fixed pacing frequency of 8 Hz, neither RMP (baseline: -76 ± 1 mV, ISO: -76 ± 1 mV, *p* = 0.89, *n* = 9), APD_90_ (baseline: 22 ± 3 ms, ISO: 16 ± 1 ms, *p* = 0.14, *n* = 9), aERP (baseline: 27 ± 12 ms, ISO: 24.0 ± 10 ms, *p* = 0.48, *n* = 9), upstroke velocity (baseline: 103 ± 24 V/s, ISO: 122 ± 32 V/s, *p* = 0.11, *n* = 9), or action potential amplitude (APA; baseline: 85 ± 7 mV, ISO: 83 ± 5 mV, *p* = 0.59, *n* = 9) were significantly affected by ISO as compared to baseline at 8 Hz pacing (**Figure 3**).

### Effects of Muscarinic Stimulation on Action Potential Parameters

Muscarinic activation resulted in drastic alterations in a number of AP parameters (**Figure 4**). Following addition of CCh, the RMP was hyperpolarized by -6 ± 1 mV. This was accompanied by shortening of APD90 (by 7 ± 2 ms, p = 0.0014, n = 11) and aERP (by 4 ± 2 ms, p = 0.078, n = 11). The upstroke velocity (baseline: 130 ± 38 V/s, CCh: 126 ± 31 V/s, p = 0.57, n = 11) and APA (baseline: 88 ± 8 mV, CCh: 89 ± 8 mV p = 0.58, n = 11), however, were not significantly affected.

As we previously have found that pharmacological inhibition of SK channels has an antiarrhythmic effect in non-stimulated isolated rat atria (Skibsbye et al., 2014), we wished to analyze whether this also was the case in β-adrenergic and muscarinic stimulated atria. Inhibition of SK channels was accomplished by applying 10 μM ICA. At this concentration, ICA efficiently blocks SK channels in multicellular atrial tissue samples, but does not block other prominent cardiac potassium ion channels (Skibsbye et al., 2014).

### SK Channel Inhibition Prolongs aERP and APD and Prevents AF During β-Adrenergic Activation

In ISO-stimulated atria, application of 10 μM ICA, after 30 min, resulted in a significantly prolonged APD_50_ (from 7 ± 1 to 10 ± 1 ms, *p* < 0.01, *n* = 5) and APD_90_ (from 17 ± 2 to 29 ± 3 ms, *p* < 0.01, *n* = 5) as well as aERP (from 22 ± 5 to 49 ± 9 ms, *p* < 0.05, *n* = 5; **Figure 5**), leading to increased post-repolarization refractoriness (Coronel et al., 2012). Furthermore, a reduced upstroke velocity was observed (from 111 ± 14 to 74 ± 12 V/s, *p* < 0.05). Even though ISO-treated atria were only susceptible to shorter runs of AF following burst pacing, ICA was still capable of suppressing the AF burden even further (from 7 ± 5 to 0.1 ± 0.1 s, *p* < 0.5, *n* = 5, **Figure 5G**).

### SK Channel Inhibition Prolongs aERP and APD and Prevents AF During Muscarinic Activation

Block of SK channels with 10 μM ICA in CCh activated atria resulted in both drastic prolongation of APD (APD_90_: from 19 ± 1 to 42 ± 5 ms, p < 0.0001, n = 6) and aERP (from 17 ± 2 to 32 ± 3 ms, p < 0.0001, n = 6; **Figure 6**). There was a trend to a less negative RMP (from -78 ± 1 to -77 ± 2 mV, p = 0.58, n = 6) and a reduced upstroke velocity (from 122 ± 6 to 98 ± 11, p = 0.71, n = 6). AF duration following ICA application was profoundly reduced (from 10 ± 7 to 2 ± 1, p < 0.05, n = 6; **Figure 6G**).

### Flecainide Prolongs aERP and APD and Prevents AF Under Muscarinic Stimulation

CCh had significant potentiating effects on AF inducibility compared to ISO. In order to benchmark the anti-AF effect of ICA toward a clinically relevant therapy, we applied the classic antiarrhythmic compound Fleca on CCh activated atria (**Figure 7**). Following application of 1 μM Fleca APD_90_ was augmented (from 14 ± 2 to 22 ± 3 ms, *p* < 0.005, *n* = 6) and aERP (from 15 ± 2 to 29 ± 3 ms, *p* < 0.001, *n* = 6), leading to post-repolarization refractoriness. Upstroke velocity was decreased from 152 ± 9 to 103 ± 6 mV/s following Fleca application. Application of Fleca almost completely suppressed any induction of AF following burst pacing (from 38 ± 15 to 1 ± 1 s, *p* < 0.05, *n* = 6). Antiarrhythmic efficacy (expressed as percentage shortening of mean AF episodes) was not different between ICA and flecanide (88 ± 24% vs. 98 ± 41%, n.s. unpaired *t*-test), indicating that the two compounds exhibit similar efficacies at their respective free exposures.

## Discussion

β-adrenergic and muscarinic activation of rat right atrium was investigated to evaluate the changes in electrophysiological parameters which could affect arrhythmia induction, maintenance and termination. Following, pharmacological intervention with ICA and Fleca was performed to address the antiarrhythmic potential of these compounds targeting primarily SK channels and Na^+^ channels, respectively.

Carbachol activation resulted in immediate abbreviations of APD and aERP, and a hyperpolarized RMP, while no significant changes, besides increased beating frequency, were observed for ISO activated atria.

### Arrhythmia Duration Following Burst Pacing

Short runs of AF could be triggered in almost all experiments following burst pacing for ∼1 s. During arrhythmias, take-off potential (minimal diastolic potential) prior to the next upstroke was depolarized to around ≈-65 mV in contrast to ≈-75 mV during SR. This 10 mV shift has profound effect on Na^+^ channel availability, due to increased voltage-dependent inactivation (Petitprez et al., 2008). This effect is observed by a drastic decrease in upstroke velocity, which is expected to provoke a reduction in conduction velocity (Skibsbye et al., 2015). ISO activated atria had shorter AF durations compared to both basal and CCh stimulated atria. The minor increase in duration and frequency of atrial arrhythmias in CCh activated atria did not reach significance as compared to non-stimulated (basal) control atria. Recently, Zafalon et al. (2013), who also investigated isolated rat atria, found a minor, but significant, increase in the frequency of atrial tachycardia following CCh incubation.

Increase of both sympathetic and vagal activation can potentially increase the risk of atrial arrhythmias (Chen et al., 2014; Hou et al., 2016), and in particular induction of arrhythmias has been found to be promoted by vagal stimulation with ACh or CCh (Goldberger and Pavelec, 1986; Godoy et al., 1999; Zafalon et al., 2004; Kirchhoff et al., 2015; Skibsbye et al., 2015). The changes in electrophysiological parameters during muscarinic activation lead to opening of the K_ir_3.1/K_ir_3.4 channel complex underlying the I_K,ACh_ current. In concordance with other studies, we find this to increase phase 3 repolarization, abbreviation of APD_90_, hyperpolarization of RMP, and a dramatic shortening of the aERP (Iijima et al., 1987; Yang et al., 1996). The CCh induced decrease in ERP will translate into a shortened wavelength (WL) of excitation propagation, promoting re-entry arrhythmias (Nattel, 2002; Comtois et al., 2005; Nattel et al., 2005). This mechanism might explain why β-adrenergic activation with ISO (where ERP was found unaltered) showed less effective in promoting AF compared to muscarinic activation. Our findings are in agreement with Liu and Nattel (1997) who reported the difference in sympathetic and vagal effects on AF in dogs, showing that sympathetic activation did not promote and maintain AF to the same extend as vagal stimulation, directly comparable with WL shortening. In addition, the authors showed that vagal, and not sympathetic activation, abbreviated the atrial refractoriness (Liu and Nattel, 1997).

The cellular effects of CCh are profound, whereas the cellular effects of isoproterenol are minor. Despite this, at the organ level, ISO reduced both frequency and duration of AF more than CCh. This might be an important finding, as it suggests that the changes of the cellular parameters are not causal for the antiarrhythmic efficacy.

In the study, we did not detect a significant difference in AF duration between CCh-treated and non-treated atria. The reason for this may be explained by the additional effect of activating I_K,Ach_, as activation of this current will also, in addition to shorten APD_90_ and ERP, shift RMP toward more hyperpolarized potentials. RMP hyperpolarization releases a larger fraction of voltage-dependently inactivated sodium channels, which on one the hand increases cellular excitability, but on the other hand speeds up tissue conduction velocity, prolonging the functional WL. This indirect augmented WL could be speculated to be a reason for CCh treated atria not translating into more proarrhythmic substrates, as one would have expected.

### SK Channel Inhibition on β-Adrenergic and Muscarinic Activated Atrium

In order to investigate the effect on activated atria, the SK channel blocker ICA was applied. SK channel inhibition showed significant antiarrhythmic effects in this model, both in the presence of β-adrenergic and muscarinic activators, showing a prolongation of both APD and aERP. These findings on AP parameters thereby complement previous studies (Diness et al., 2010; Skibsbye et al., 2014, 2015; Kirchhoff et al., 2015) providing further evidence for a mechanistic understanding of SK currents in AF, where a slowing to the AP repolarization seems to be a pivotal parameter. It has previously been shown that SK channel blockers result in depolarization of RMP – suggested to have indirect inhibitory effects on sodium conductance due to voltage-dependent inactivation (Skibsbye et al., 2014, 2015). However, the depolarization following application of ICA trended toward, but did not reach, significant levels in this study. However, we did observe a reduction in conduction velocity in the ISO-treated group following ICA application and a trend toward a reduction in the CCh treated group following ICA application. Since 10 μM ICA is not expected to block the cardiac sodium current (Skibsbye et al., 2014), these results indicate that the slight shift in depolarization of the RMP affects sodium channel availability and thereby upstroke velocity.

Interestingly, in the CCh treated atria, SK channel inhibition demonstrated antiarrhythmic properties with similar efficacy to the antiarrhythmic class IC drug Fleca. Due to the primary target of Fleca being Nav1.5, post-repolarization refractoriness was observed, suggesting this to be the primary driver for the antiarrhythmic efficacy representing a classic class IC AAD. In the same setting, ICA application caused extensive APD prolongation exceeding tissue refractoriness, here representing a more classical Class III AAD. The antiarrhythmic effects reported here highlight the possibility of SK channel inhibition as being efficacious also in clinical AF.

### Limitations

Sympathetic and vagal activation was limited to pharmacological interventions in the isolated atria, since vagal and sympathetic nerve innervation are blunted. ISO stimulation makes the atria contract much more rigorously. Maintaining proper sealing to the cardiomyocytes thereby becomes challenging, resulting in a low success-rate with ISO (≈20%). Due to this limited number of achievable experiments with ISO, it was decided only to benchmark Fleca vs. ICA efficacy in the setting of vagal stimulation. All experiments apart from the groups treated with ISO were performed at a pacing frequency of 5 Hz in order to overcome the atrial sinus frequency. As ISO treatment increases the beating frequency, these experiments were performed at 8 Hz. Comparison between these groups should therefore be done with caution as frequency is an important parameter in regulating AP morphology and arrhythmogenesis. None of the AADs tested in this model are absolute selective for their primary target at the concentrations tested in this study. The sodium-channel blocker Fleca and the SK channel blocker ICA are promiscuous and inhibit, e.g., other potassium channels to some extent. Therefore, it is likely that the antiarrhythmic effects, including the effects on conduction velocity, observed to some degree reflect the inhibitory effects of multiple targets.

## Conclusion

Vagal activation with CCh leads to shortening of atrial APD and ERP and hyperpolarization of RMP compared to control atria. β-adrenergic activated atria did not alter AP parameters compared to control but resulted in reduced arrhythmia duration and frequency. In vagally stimulated atria, pharmacological inhibition of SK channels by ICA demonstrates similar antiarrhythmic efficacy to the sodium channel inhibitor Fleca with both compounds prolonging the APD and aERP. Furthermore, SK channel inhibition reduced the duration of arrhythmia in β-adrenergic activated atria, altogether demonstrating this as an interesting antiarrhythmic target in variable autonomic imbalanced scenarios. The possible clinical implication of SK blockers for the treatment of AF in hyper-vagal and hyper-sympathetic patients calls for further investigation.

## Author Contributions

TJ and LS: hypothesis. TJ, LS, AU-N, and AB: experimental design. AU-N and AB: daily refinement of experimental details. LS, KB, AU-N, and AB: data handling. TJ, TC, LS, and KB: data analyses. TJ, LS, AU-N, AB, KB, and TC: writing the MS.

## Conflict of Interest Statement

The authors declare that the research was conducted in the absence of any commercial or financial relationships that could be construed as a potential conflict of interest.

## Abbreviations

aERP: atrial effective refractory period
AF: atrial fibrillation
AP: action potential
APD: action potential duration
CCh: carbachol
Fleca: flecainide
ICA: *N*-(pyridine-2-yl)-4-(pyridine-2-yl)thiazol-2-amine
ISO: isoprenaline (isoproterenol)
MAP: monophasic action potential
RMP: resting membrane potential
SK: small conductance Ca^2+^-activated K^+^
